# MiR-26b-5p inhibits cell proliferation and EMT by targeting MYCBP in triple-negative breast cancer

**DOI:** 10.1186/s11658-021-00288-3

**Published:** 2021-12-11

**Authors:** Sugang Ma, Hui Wei, Chunyan Wang, Jixia Han, Xiumin Chen, Yang Li

**Affiliations:** 1 Department of Breast Surgery, Jinan Sixth People’s Hospital, Jinan, 250200 Shandong China; 2 Department of Obstetrics, Jinan Sixth People’s Hospital, Jinan, 250200 Shandong China; 3 Department of Laboratory Medicine, Jinan Sixth People’s Hospital, No. 1920 Huiquan Road, Zhangqiu District, Jinan, 250200 Shandong China

**Keywords:** Triple-negative breast cancer, miR-26b-5p, MYCBP, EMT

## Abstract

**Background:**

The study was designed to elucidate the association and functional roles of miR-26b-5p and c-MYC binding protein (MYCBP) in triple-negative breast cancer (TNBC).

**Method:**

Luciferase reporter assay was used to confirm the relationship between miR-26b-5p and MYCBP in TNBC cells. The expression levels of miR-26b-5p and MYCBP in tissue specimens and cell lines were determined using reverse transcription-quantitative PCR. Cell proliferation, migration and invasion were assessed using CCK-8 assay, colony formation and transwell assay.

**Results:**

We first observed that miR-26b-5p directly targets the 3′-UTR of MYCBP to inhibit MYCBP expression in MDA-MB-468 and BT-549 cells. The expression of miR-26b-5p was inversely correlated with MYCBP expression in TNBC tissues. We further demonstrated that MYCBP knockdown suppressed the proliferation, migration and invasion of TNBC cells. Furthermore, MYCBP overexpression counteracted the suppressive effect of miR-26b-5p on TNBC cell behaviors. Western blot analysis demonstrated that the E-cadherin protein level was increased, while protein levels of N-cadherin and vimentin were decreased in cells transfected with miR-26b-5p, which were all reversed by ectopic expression of MYCBP.

**Conclusions:**

In summary, our findings revealed the tumor suppressive role of miR-26b-5p in regulating TNBC cell proliferation and mobility, possibly by targeting MYCBP.

## Background

Triple-negative breast cancer (TNBC) as the most malignant subtype of breast cancer is characterized by lack of estrogen receptor (ER), progesterone receptor (PR) and human epidermal growth factor receptor 2 (HER2) [1]. TNBC is more aggressive and has a worse prognosis compared with other subtypes of breast cancer, which is associated with few effective targeted therapies [2, 3]. Thus, there is always high demand for investigating novel molecular factors that influence growth and metastasis of TNBC cells for the enrichment of therapeutic strategies.

MicroRNAs (miRNA/miRs) are non-coding single-stranded small molecule RNAs with a length of 20 to 24 nucleotides that usually act as vital regulators in cellular processes, including proliferation, differentiation and apoptosis [4, 5]. Accumulating evidence has indicated that aberrant expression of miRNAs is correlated with the initiation and progression of TNBC [6–8]. In recent years, miR-26b-5p has frequently been reported as downregulated in several cancers, including bladder cancer [9], hepatocellular carcinoma [10] and thyroid cancer [11]. Further studies have been performed on the molecular functions of miR-26b-5p in various types of tumor cells. For example, miR-26b-5p acts as a tumor suppressor through suppressing cell proliferation and inducing cell apoptosis in multiple myeloma [12]. MiR-26b-5p inhibits proliferation, migration, invasion and epithelial-mesenchymal transition (EMT) in human papillary thyroid cancer [13]. It was also reported that miR-26b-5p exerts suppressive effects on cell proliferation in lung adenocarcinoma cells [14]. Notably, miR-26b was recently reported to be downregulated in locally advanced and inflammatory breast cancer [15]. Moreover, miR-26b impairs viability and triggers apoptosis of human breast cancer cells [16]. However, the functional role and the underlying mechanism of miR-26b-5p in the progression of TNBC have remained largely unknown.

A previous study reported that c-MYC binding protein (MYCBP) binds to the N-terminus of the oncogenic protein c-MYC through its C-terminal structure to promote tumorigenesis [17]. Activation of the MYC gene is frequently associated with the malignant properties of tumors, including increased mobility, and invasive and metastatic capacities [18]. It is well documented that the MYCBP/c-MYC pathway plays an important role in the development of TNBC [19]. In our earlier work, MYCBP was the target gene for miR-26b-5p based on the TargetScan prediction, which made us hypothesize that miR-26b-5p may play an important role in TNBC cell proliferation, migration and invasion by targeting MYCBP.

To validate our hypothesis, we first investigated the association between miR-26b-5p and MYCBP in TNBC cells. Then, we further evaluated the function of miR-26b-5p and MYCBP in the malignant behaviors of TNBC cells and characterized the underlying mechanism. Our work may provide novel insights into the biological significance of the miR-26b-5p/MYCBP axis in TNBC.

## Materials and methods

### Cell culture and transfection

Two human TNBC cell lines, MDA-MB-468 and BT-549, were purchased from the American Type Culture Collection (Manassas, VA, USA) and cultured in RPMI-1640 medium supplemented with 10% FBS (Gibco, Grand Island, NY, USA) in a humidified 5% CO_2_ incubator at 37 °C. The miR-26b-5p mimics, miR-26b-5p negative control (miR-NC), miR-26b-5p inhibitor, inhibitor NC, small interfering RNAs for knockdown of MYCBP (si-MYCBP^#^1 and si-MYCBP^#^2) and a negative control (si-NC) were commercially obtained from GenePharma Co. Ltd. (Shanghai, China). The MYCBP overexpression plasmid was constructed by amplifying the complementary DNA (cDNA) fragment of MYCBP via PCR and inserted into the pcDNA3.1 vector, Sigma-Aldrich Co., LLC (St. Louis, MO, USA). The transfection assay was conducted with Lipofectamine 2000 (Invitrogen, Carlsbad, CA, USA).

### Luciferase reporter assay

The MYCBP 3′-UTR fragment containing the wild-type (WT) or mutant (MUT) miR-26b-5p binding sites were synthesized and inserted into the pGL3 Basic vector (Promega, Madison, Wisconsin). MDA-MB-468 and BT-549 cells were co-transfected with 200 ng of WT or MUT MYCBP recombinant plasmid and 100 ng of miR-26b-5p mimics or miR-NC (miR-26b-5p inhibitor or inhibitor NC) with Lipofectamine 2000 for 48 h. Afterwards, transfected cells were harvested and the luciferase activities of firefly and *Renilla* were determined using the Dual Luciferase Reporter Assay Kit (Promega). The relative luciferase activity was calculated as the ratio of firefly luciferase activity versus *Renilla* luciferase activity.

### Clinical specimens

A total of 60 freshly frozen TNBC tissues and 26 adjacent nontumor tissues (ANT, > 5 cm away from the tumor tissues), which included 20 pairs of TNBC tissues and matched ANT from the same patients, were obtained from Jinan Sixth People's Hospital (Shandong, China). Written informed consent was obtained from all patients included in the present study. None of the patients (age range 33–64 years) received prior chemotherapy or radiotherapy. All collected specimens were stored at − 80 °C until further use. This study was approved by the ethics committee of Jinan Sixth People's Hospital (Shandong, China).

### Reverse transcription-quantitative PCR

Total RNA was extracted from tissues or cell lines using Trizol reagent (Thermo Fisher Scientific, Waltham, MA, USA) and 1 µg of RNA of each sample was reversed transcribed to cDNA by a Reverse Transcription Kit (Invitrogen, CA, USA). PCR amplification was performed using 1 µg of cDNA for the SYBR Green Master mix (Invitrogen; Thermo Fisher Scientific, Inc.) on an ABI Prism7500 fast real-time PCR system (Applied Biosystems, Foster City, CA) with the cycling conditions consisting of initial denaturation at 95 °C for 30 s, followed by 42 cycles of amplification at 95 °C for 5 s and 60 °C for 20 s. The relative expression of miR-26b-5p and MYCBP was normalized to U6 and β-actin, respectively, using the 2^−ΔΔCt^ method.

### Cell Counting Kit‐8 (CCK‐8) assay

Transfected cells were seeded into 96-well plates at a density of 3,000 cells per well in 100 μL of culture medium and incubated at 37 °C for 0, 24, 48 or 72 h. Subsequently, cells in each well were incubated with 10 μL of CCK-8 reagent (Beyotime, Shanghai, China). After being maintained for another 2 h at 37 °C, the optical density (OD) values were measured at 450 nm using a microplate reader (Bio-Tek, Winooski, USA). The experiment was performed in triplicate.

### Colony formation assay

Briefly, transfected cells at a density of 500 cells per well were seeded into six-well plates and cultured for 10–14 days in a sterile incubator with 5% CO_2_ at 37 °C. The colonies were fixed with ice-cold methanol at 4 °C and stained with 0.5% crystal violet for 30 min at room temperature. Images were captured using a digital camera in order to assess the number of stained cell colonies (containing at least 50 cells per colony).

### Cell migration and invasion assay

For the cell migration assay, approximately 2 × 10^5^ transfected cells suspended in 200 µL of serum-free medium were added to the upper transwell chambers (pore size, 8 μm; Corning Inc., Tewksbury, MA, USA). Meanwhile, the lower chambers were filled with 600 µL of complete culture medium. After 24 h of incubation, the cells in the bottom chamber were collected, fixed with 4% paraformaldehyde, and stained with 0.1% crystal violet. Finally, the cells were visualized using a light microscope (Olympus Corporation; magnification, × 100), and their number was estimated by manual counting. The procedures of invasion assay were similar to the migration assay except that the upper chamber was precoated with diluted Matrigel (final concentration of 250 μg/ml/well, Corning).

### Western blot analysis

The total protein sample was extracted using RIPA lysis buffer (Thermo Fisher Scientific, Waltham, MA, USA) and the protein concentration was determined by a BCA Protein Assay Kit (Beyotime) according to the protocol. A total of 30 µg of protein/lane was separated by 10% SDS-PAGE gels and then transferred onto polyvinylidene difluoride (PVDF) membranes (Millipore, Billerica, MA, USA). After blocking with 5% nonfat skimmed milk for 2 h at room temperature, the membranes were incubated with primary antibodies against MYCBP (ab86078; host, rabbit; 1:2000; Abcam), E-cadherin (Ab-171; host, rabbit; 1:1000; Proteintech), N-cadherin (#4061; host, rabbit; 1:1000; Cell signaling Technology), vimentin (#5741; host, rabbit; 1:1000; Cell Signaling Technology) and β-actin (ab8229; host, goat; 1:5000; Abcam) overnight at 4 °C, followed by incubation with anti-rabbit antibody conjugated to horseradish peroxidase secondary antibodies (#7074; host, goat; 1:5000, Cell Signaling Technology) for 2 h at room temperature. Protein bands were visualized by enhanced chemiluminescence (Millipore) according to the manufacturer’s protocol. β-actin served as the loading internal reference.

### Statistical analysis

All statistical analyses were performed using GraphPad Prism 6.0 software and data were presented as mean ± standard deviation (SD). Spearman’s correlation analysis was conducted to assess the correlation between miR-26b-5p and MYCBP. Two-tailed Student’s t test was used to determine the differences between two groups, while one-way ANOVA followed by the Tukey test was utilized to assess the differences among more than two groups. The values of *p* less than 0.05 were considered to be statistically significant.

## Results

### MiR-26b-5p directly targets the 3′-UTR of MYCBP to inhibit MYCBP expression

**Fig. 1:**
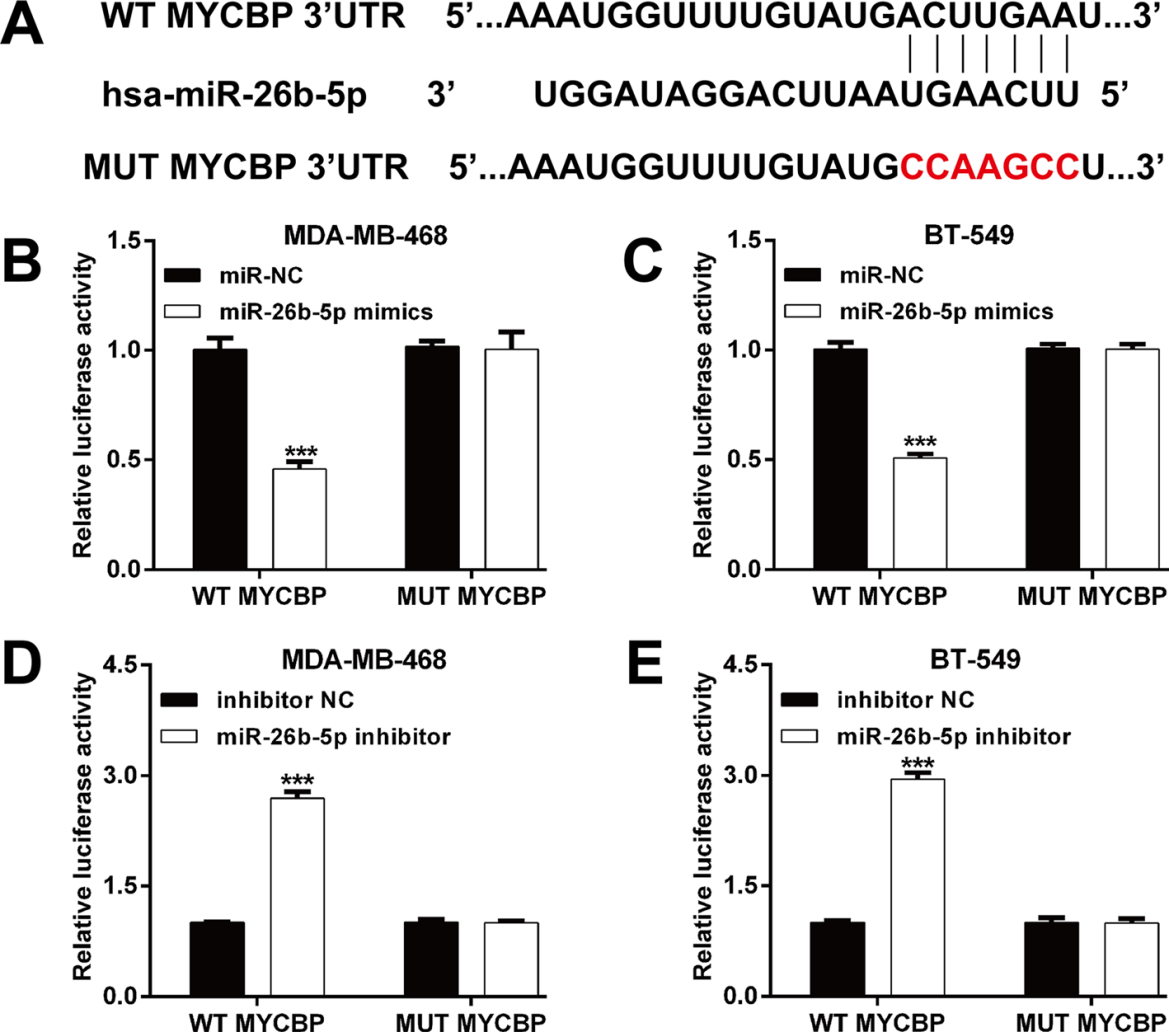
MYCBP is a direct target of miR-26b-5p. **A** The binding site for miR-940 in the 3-′UTR of FOXO3 predicted by bioinformatics was presented. Luciferase reporter assay evaluation of the interaction between miR-26b-5p and the 3′-UTR of MYCBP mRNA. Luciferase activity was determined 48 h after co-transfection of luciferase constructs containing the wild-type (WT) or a mutated (MUT) 3′-UTR of MYCBP mRNA and miR-26b-5p mimics or miR-NC in MDA-MB-468 (**B**) and BT-549 (**C**) cells. Luciferase activity was determined 48 h after co-transfection of luciferase constructs containing the WT or a MUT 3′-UTR of MYCBP mRNA and miR-26b-5p inhibitor or inhibitor NC in MDA-MB-468 (**D**) and BT-549 (**E**) cells. Data represent the mean ± SD of three independent experiments. ***p < 0.001, compared with miR-NC or inhibitor NC

**Fig. 2:**
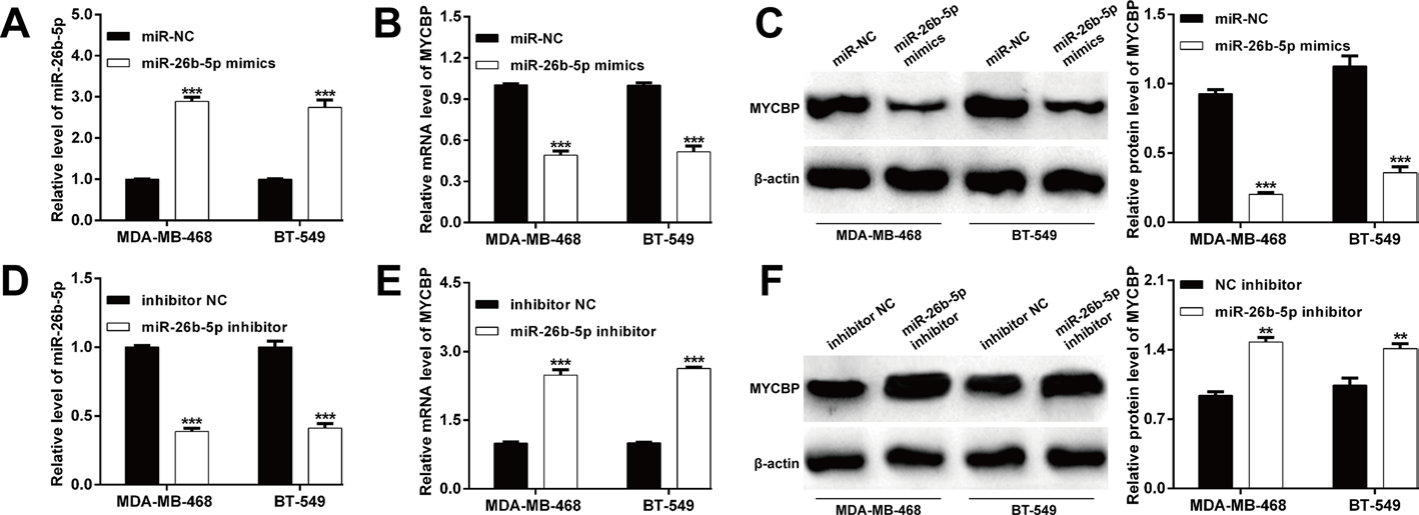
miR-26b-5p regulation of endogenous MYCBP expression. The expression levels of **A** miR-26b-5p, **B** MYCBP mRNA and **C** MYCBP protein were determined in MDA-MB-468 and BT-549 cells after transfection with miR-26b-5p mimics or miR-NC. The expression levels of **D** miR-26b-5p, **E** MYCBP mRNA and **F** MYCBP protein were determined in MDA-MB-468 and BT-549 cells after transfection with miR-26b-5p inhibitor or inhibitor NC. Data represent the mean ± SD of three independent experiments. ***p < 0.001, compared with miR-NC or inhibitor NC

According to the bioinformatics prediction that putative target sites for miR-26b-5p were located within the 3′-untranslated regions (UTRs) of MYCBP mRNA (Fig. 1A), we constructed WT and MUT MYCBP luciferase vectors to perform a luciferase reporter assay to further investigate the interaction between miR-26b-5p and MYCBP in TNBC cells. When MDA-MB-468 and BT-549 cells were co-transfected with miR-26b-5p mimics or miR-NC and WT or MUT MYCBP vectors, the miR-26b-5p mimics’ transfection significantly reduced luciferase activity by approximately 50% compared with co-transfection of WT MYCBP and miR-NC (Fig. 1B, C). In contrast, miR-26b-5p inhibitor transfection clearly increased the activity of a luciferase reporter fused to the WT 3′-UTR of MYCBP, but not of the MUT reporter in MDA-MB-468 (Fig. 1D) and BT-549 (Fig. 1E) cells. In addition, we determined how miR-26b-5p regulated endogenous MYCBP expression in TNBC cells. As expected, overexpression of miR-26b-5p by transfection of the miR-26b-5p mimics (Fig. 2A) resulted in a significant reduction of MYCBP mRNA (Fig. 2B) and protein (Fig. 2C) levels in MDA-MB-468 and BT-549 cells. Conversely, knockdown of miR-26b-5p (Fig. 2D) by transfection of the miR-26b-5p inhibitor upregulated the expression of MYCBP at the mRNA (Fig. 2E) and protein (Fig. 2F) levels in MDA-MB-468 and BT-549 cells. These results suggested that miR-26b-5p negatively regulated the expression of MYCBP through its 3′UTR region binding sequence.

### MiR-26b-5p was downregulated, while MYCBP was upregulated in TNBC tissues

**Fig. 3:**
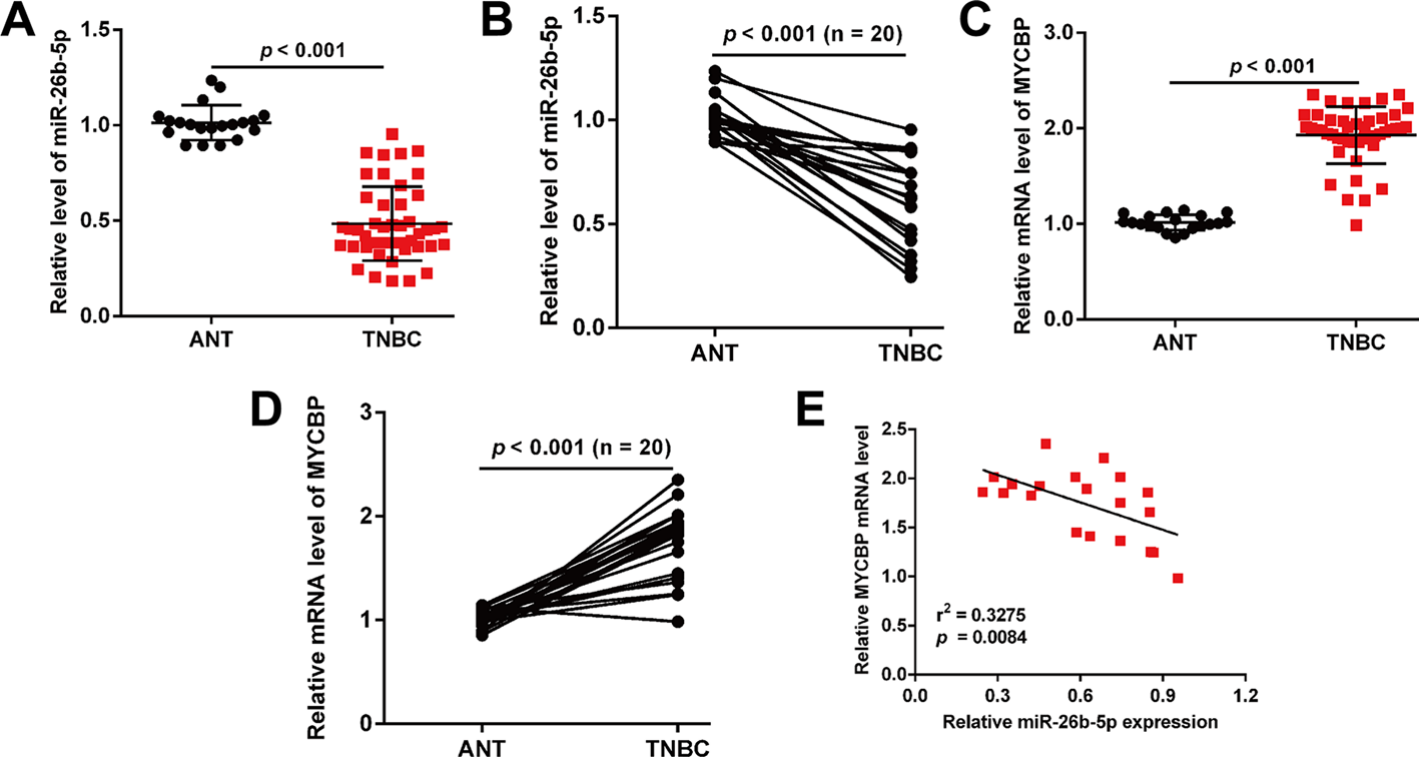
Expression of miR-26b-5p and MYCBP in TNBC patients. **A** MiR-26b-5p levels were significantly lower in TNBC tissues (n = 60) than in adjacent nontumor tissues (ANT, n = 26). **B** The expression of miR-26b-5p in 20 pairs of TNBC tissues and matched ANT was determined using PCR assay. **C** MYCBP levels were significantly higher in TNBC tissues (n = 60) than in ANT (n = 26). **D** The expression of MYCBP in 20 pairs of TNBC tissues and matched ANT was determined using PCR assay. **E** The correlation between miR-26b-5p and MYCBP expression in TNBC tissues was determined by Spearman’s correlation analysis

Reverse transcription-quantitative PCR was performed to determine the expression of miR-26b-5p and MYCBP in TNBC tissues and adjacent non-tumor tissues. As shown in Fig. 3A, the expression of miR-26b-5p was significantly downregulated in TNBC tissues (n = 60), compared with adjacent non-tumor tissues (n = 26). Consistently, miR-26b-5p levels were found to be much lower in TNBC tissues by detecting its expression in 20 pairs of TNBC tissues and matched adjacent nontumor tissues (Fig. 3B). As depicted in Fig. 3C, PCR assay revealed that the expression of MYCBP mRNA in TNBC tissues was dramatically higher than adjacent nontumor tissues. Similar results were observed in 20 pairs of TNBC tissues and matched adjacent nontumor tissues (Fig. 3D). Moreover, we observed a significant negative correlation between miR-26b-5p and MYCBP expression in TNBC tissues (Fig. 3E, R^2^ = 0.3275, *p* = 0.0084).

### Knockdown of MYCBP suppressed the proliferation, migration and invasion of TNBC cells

**Fig. 4:**
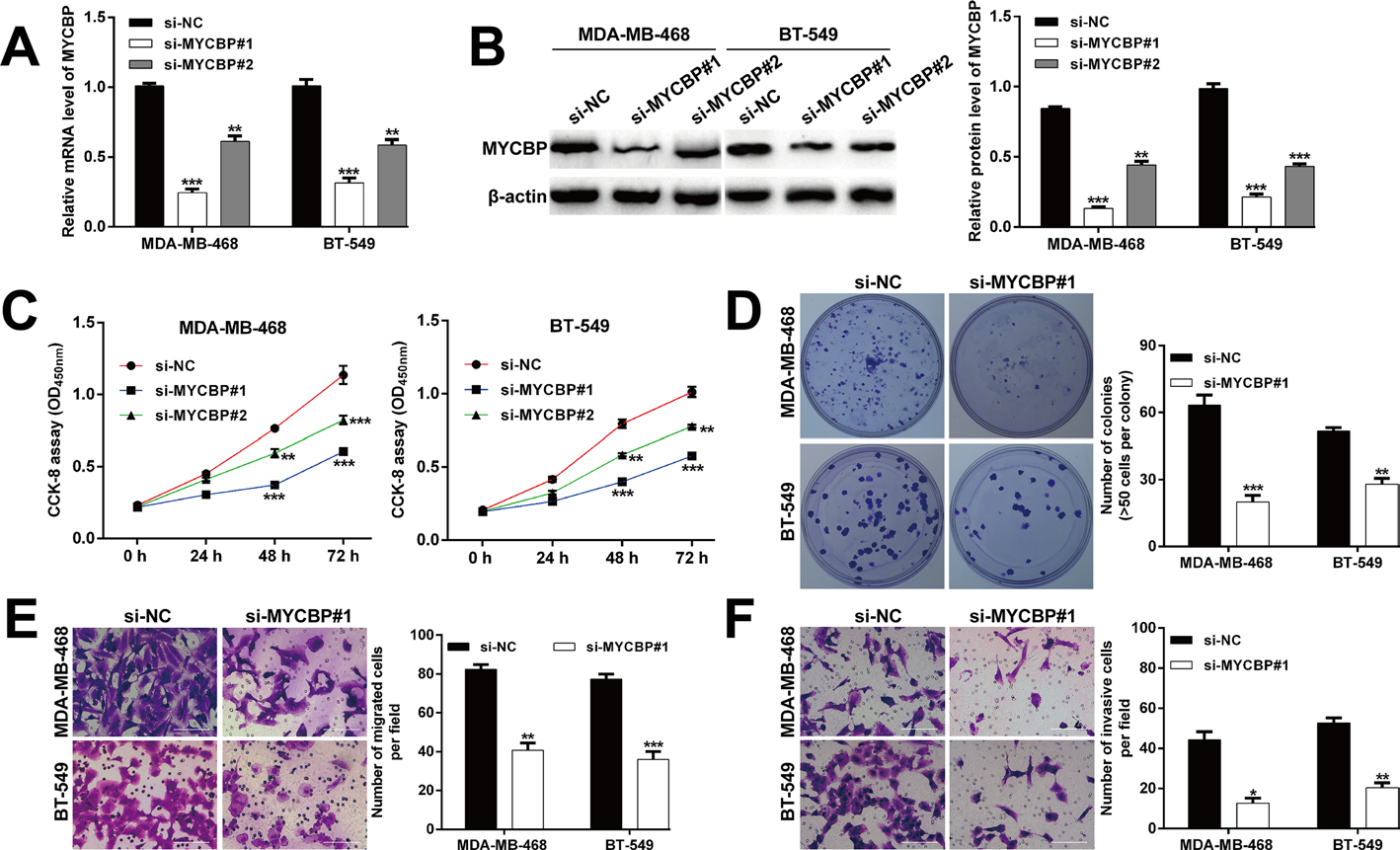
Silencing of MYCBP inhibited TNBC cell proliferation, migration and invasion. Transfection efficiency of si-MYCBP^#^1 or si-MYCBP^#^2 in MDA-MB-468 and BT-549 cells was detected by PCR assay (**A**) and western blot analysis (**B**). **C** CCK-8 assay was used to determine the cell viability of MDA-MB-468 and BT-549 cells after transfection with si-MYCBP^#^1, si-MYCBP^#^2 or si-NC. **D** The influence of MYCBP knockdown on cell proliferation of MDA-MB-468 and BT-549 cells was detected by colony formation assay. MYCBP knockdown decreased the number of migrated (**E**) and invasive (**F**) cells. Magnification, ×200; scale bar, 100 μm; data are shown as mean ± SD (n = 3). *p < 0.05, **p < 0.01 and ***p < 0.001 compared with si-NC group

Considering the upregulation of MYCBP in TNBC, we performed loss-of-function assays in TNBC cell lines to investigate the functional role of MYCBP in MDA-MB-468 and BT-549 cells transfected with siRNAs targeting MYCBP (si-MYCBP^#^1 or si-MYCBP^#^2). Reverse transcription-quantitative PCR (Fig. 4A) and western blot analysis (Fig. 4B) were first used to confirm the obvious decrease of MYCBP mRNA and protein expression. CCK-8 assay showed that the cell viability of MDA-MB-468 and BT-549 cells was significantly inhibited by siRNA-mediated downregulation of MYCBP (Fig. 4C). Notably, si-MYCBP^#^1 presented stronger suppressive effects on MYCBP expression and cell viability; it was thus selected for the subsequent experiments. The colony formation assay revealed that si-MYCBP^#^1 transfection reduced the number of colonies in MDA-MB-468 and BT-549 cells (Fig. 4D). Additionally, the migratory (Fig. 4E) and invasive (Fig. 4F) abilities of MDA-MB-468 and BT-549 cells were remarkedly inhibited by MYCBP knockdown. These data suggested that MYCBP might contribute to malignant phenotypes of TNBC in vitro.

### MYCBP overexpression counteracted the suppressive effect of miR-26b-5p on TNBC cell behaviors

**Fig. 5:**
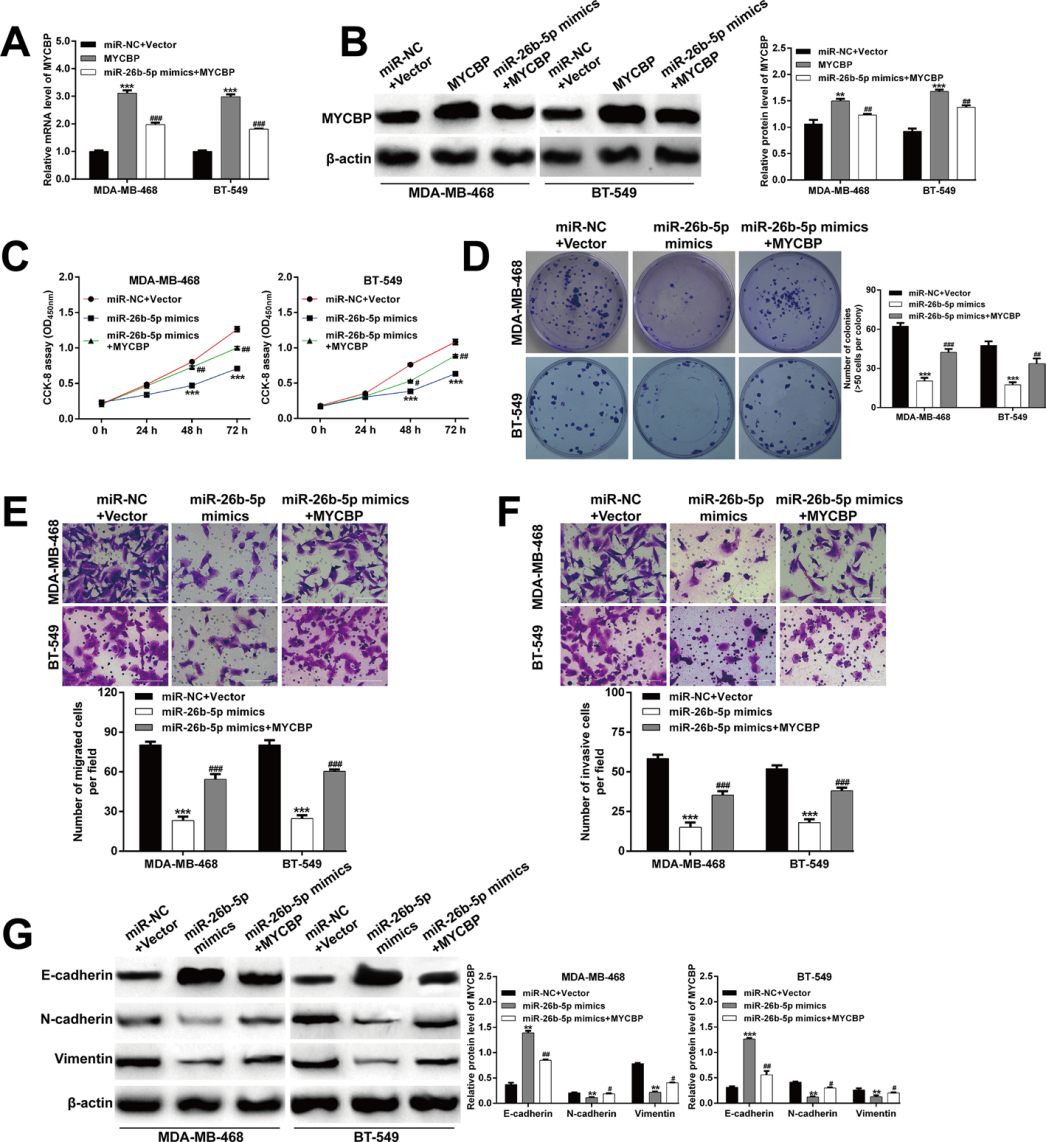
MYCBP overexpression counteracted the suppressive effect of miR-26b-5p on TNBC cell behaviors. MDA-MB-468 and BT-549 cells were co-transfected with MYCBP overexpressing plasmid and miR-26b-5p mimics. The expression levels of MYCBP mRNA (**A**) and protein (**B**) in MDA-MB-468 and BT-549 cells after indicated transfection were determined by PCR and western blot assays. **C** Cell viability was determined using CCK-8 assay in MDA-MB-468 and BT-549 cells after indicated transfection. **D** The number of colonies in MDA-MB-468 and BT-549 cells after indicated transfection. Cell migration (**E**) and invasion (**F**) were assessed by transwell assay in MDA-MB-468 and BT-549 cells after indicated transfection. Magnification, ×200; scale bar, 100 μm; data are shown as mean ± SD (n = 3). ***p < 0.001 compared with miR-NC + Vector group; ^#^*p* < 0.05, ^##^*p* < 0.01, ^###^*p* < 0.001 compared with MYCBP or miR-26b-5p mimics; **G** The protein levels of E-cadherin, N-cadherin and vimentin were measured by western blot analysis in MDA-MB-468 and BT-549 cells after indicated transfection

We next examined whether miR-26b-5p negatively regulated MYCBP to suppress TNBC cell proliferation, migration, and invasion. MDA-MB-468 and BT-549 cells were co-transfected with MYCBP overexpressing plasmid and miR-26b-5p mimics. The transfection efficiency of MYCBP overexpression plasmid was first detected in TNBC cells. As shown in Fig. 5A, B, the expression levels of MYCBP mRNA and protein were significantly upregulated in MDA-MB-468 and BT-549 cells after transfection with the MYCBP overexpression plasmid, while they could be attenuated by miR-26b-5p mimics. The cell proliferation was examined using CCK-8 assay and colony formation. We found that the cell viability (Fig. 5C) and colonies (Fig. 5D) in co-transfection groups were dramatically higher than in cells transfected with miR-26b-5p mimics in MDA-MB-468 and BT-549 cells. Compared with cells after transfection with miR-26b-5p mimics, the number of migratory (Fig. 5E) and invasive (Fig. 5F) cells was dramatically increased in cells co-transfected with MYCBP overexpressing plasmid and miR-26b-5p mimics. Furthermore, the E-cadherin protein level was increased, while protein levels of N-cadherin and vimentin were decreased in cells transfected with miR-26b-5p, which were all reversed by ectopic expression of MYCBP in MDA-MB-468 and BT-549 cells (Fig. 5G). Taken together, these results indicated that MYCBP was a downstream target of miR-26b-5p, which could antagonize miR-26b-5p-mediated inhibitory effects on TNBC cell proliferation and the EMT process.

## Discussion

In our study, we first confirmed that miR-26b-5p negatively regulated the expression of MYCBP through its 3′UTR region binding sequence in TNBC cells. Moreover, miR-26b-5p was downregulated, while MYCBP was upregulated in TNBC tissues compared with adjacent nontumor tissues. The expression of miR-26b-5p was inversely correlated with MYCBP in TNBC tissues. These findings preliminarily indicated that miR-26b-5p might be a tumor suppressor by targeting MYCBP in TNBC. Similarly, decreased miR-26b-5p expression has frequently been reported in bladder cancer [9], hepatocellular carcinoma [10] and thyroid cancer [11]. A recent study by Wilke et al. [20] indicated that the upregulation of miR-26b-5p in radiation-association breast cancer tissue samples might further demonstrate the association between miR-26b-5p and TNBC pathogenesis. In line with our data, the expression of MYCBP was highly increased in the TNBC group compared to that in the non-TNBC group [21] and also significantly overexpressed in astrocytomas in comparison with normal brain [22].

Further studies revealed that MYCBP knockdown imitated the suppressive effects of miR-26b-5p overexpression on TNBC cell proliferation, migration and invasion. Conversely, MYCBP overexpression counteracted the suppressive effect of miR-26b-5p on TNBC cell behaviors. In line with our data, miR-26b-5p inhibits proliferation, migration and invasion in human papillary thyroid cancer [13], as well as exerting suppressive effects on cell proliferation in lung adenocarcinoma [14], multiple myeloma [12] and bladder cancer [23]. The overexpression of MYCBP promoted the migration and invasion of gastric cancer cells, and vice versa when inhibited [24]. Oncogenic MYCBP could protect hepatocellular carcinoma cells against sorafenib-induced apoptosis [25]. Here, our data indicated that MYCBP is a target of tumor suppressive miR-26b-5p in TNBC cells. In a similar manner, Jiang et al. [26] found that MYCBP was one target of the tumor suppressor miR-22 in acute myeloid leukemia. Fang et al. [27] reported that MYCBP could reverse the biological effects of miR-429 on proliferation and clone formation in nephroblastoma cells. In addition, MYCBP is a target of miR-574-5p in colorectal cancer [28], miR-145 in ovarian cancer [29] and miR-451a in lung cancer [30]. Moreover, cell adhesion, proliferation, migration and invasion are involved in the EMT process and correlate with tumor metastasis [31]. Thus, we determined the regulatory role of the miR-26b-5p/MYCBP axis in the EMT process in TNBC cells. The results were consistent with our hypothesis that miR-26b-5p impaired the EMT process by targeting MYCBP, as reflected by increased E-cadherin expression and decreased expression levels of N-cadherin and vimentin in two TNBC cell lines. These results may account for the suppression of proliferation and mobility induced by miR-26b-5p overexpression or MYCBP knockdown in TNBC cells. In fact, Wang et al. [32] reported that miR-26b-5p dramatically suppressed EMT and the invasion ability of hepatocellular carcinoma cells in vitro. Zhou et al. [11] also demonstrated that miR-26b-5p inhibits proliferation, migration, invasion and EMT by degrading β-catenin in papillary thyroid cancer cells. The direct regulatory effect of MYCBP on induction of EMT has been illustrated in colorectal cancer [33] and lung cancer [30]. These data further supported our finding that MYCBP is a crucial downstream target of miR-26b-5p in TNBC cells.

## Conclusions

In summary, we demonstrated for the first time that MYCBP was a downstream gene of miR-26b-5p in TNBC cells and miR-26b-5p exerted suppressive effects on TNBC cells, possibly by targeting MYCBP. Our data indicated that the miR-26b-5p/MYCBP axis might serve as a possible therapeutic target for developing improved therapeutic approaches for TNBC patients.

## Data Availability

All data generated and analyzed during the present study are included in this article.

## Abbreviations

TNBC: Triple-negative breast cancer
MYCBP: C-MYC binding protein
3′-UTRs: 3′-Untranslated regions
siRNAs: Small interfering RNAs
cDNA: Complementary DNA
CCK-8: Cell Counting Kit-8
OD: Optical density
WT: Wild-type
HRP: Horseradish peroxidase
